# Dynamic assessment of self-regulated learning in preschool

**DOI:** 10.1016/j.heliyon.2022.e10035

**Published:** 2022-08-01

**Authors:** Janete Silva Moreira, Paula Costa Ferreira, Ana Margarida Veiga Simão

**Affiliations:** CICPSI, Faculty of Psychology, University of Lisbon, Alameda da Universidade, 1649-013 Lisbon, Portugal

**Keywords:** Self-regulated learning, Preschool, Dynamic assessment, Ecologic measures

## Abstract

The assessment of self-regulated learning is a relevant research topic in early childhood development. However, there are few ecologic measures to assess self-regulated learning in preschool as a dynamic and multidimensional process. This study aims to fill this gap by presenting the development and validation of the Dynamic Assessment of Self-regulated learning in Preschool (DASP) method. A dynamic assessment of the construct may constitute an important contribution as it enables the acquisition of cross observational, verbal, and performance data. The DASP method was developed within a theoretical framework of self-regulation, including all cyclical phases, namely, forethought, performance, and self-reflection. Specifically, this method requires children to be questioned in the forethought and self-reflection phases, and observed in the performance phase, as the researcher notes their strategies. This method is used while children engage in authentic preschool tasks. To achieve the study's aim, 214 preschool children were asked to participate. In this research, children performed the Clown task (cognitive task) and the Head-Toes-Knees-Shoulders task (motor task). Item Response Theory analyses provided good item fit for the DASP method (forethought: .99, performance: 1.00, self-reflection: .99), good values of the tasks' reliability (Clown: .92; HTKS: .85), and evidence of the participants' difficulty level in completing the tasks. Results indicated that the children experienced more difficulty in the performance phase, as opposed to the other phases. The potentialities, constraints, and practical implications of the DASP method will be discussed in terms of contributions for theory and practice.

## 1 Introduction

### 1.1 The role of self-regulated learning in preschool

Although relevant work has been conducted on the development of self-regulated learning (SRL) with older learners (e.g., Chaves-Barboza et al., 2015; Chaves-Barboza et al., 2016), less research has focused on young learners. Consequently, the pertinence of improving SRL in preschool children proved by some studies in the last decades (e.g., De la Fuente and Diaz, 2010; Ponitz et al., 2008; Whitebread et al., 2009) needs deeper investigation.

The SRL can be defined as a cyclical process of action, is continuously open to new improvements, with different progress and retreat moments and is enhanced by previous experience (Zimmerman, 2013). Accordingly, in this process, students are cognitively, metacognitively, motivationally and emotionally engaged in their learning process at varying degrees, and may be influenced by personal, contextual, and behavioral variables. The author (Zimmerman, 2013) explains that self-regulated learners tend to apply specific processes that transform their preexisting abilities into task-related behavior, not only in terms of school contents, but in different areas of functioning. This socio-cognitive perspective of SRL includes three phases, namely, forethought, performance (volitional control), and self-reflection, which reciprocally interact with each other. Moreover, key metacognitive processes, such as the use of task-related strategies, imagery, and verbal self-instruction were considered in the model's conceptualization, along with cognitive processes, such as planning and goal setting, to organize and transform information more effectively (Graham and Harris, 1989). Furthermore, motivational variables, such as self-efficacy, were also included to explain the motivational engagement that is essential to SRL (Bandura and Schunk, 1981).

Positive relations have been established between the promotion of SRL competencies in early ages and the successful management of school challenges (McClelland and Cameron, 2011; Piscalho and Veiga Simão, 2014). The recent social and health global changes are also drawing attention for the need to support the preschool education system (Munastiwi and Puryono, 2021). The SRL competencies, when exercised appropriately and timely, can contribute to children's emotional, social, cognitive, and motivational maturity (Bronson, 2000). The development of such psychological dimensions is related to the growing metacognitive awareness that can be promoted through educational practices and opportunities given to children in daily activities. SRL is included in a set of essential competencies focused on implementing competence-oriented education, problem solving, critical thinking, and the ability to cooperate, where autonomy and responsibility play an important role in our quickly changing society. Preschool is, therefore, a privileged context for the development of SRL competencies, to update assessment and validation methods and tools, and for introducing new and innovative forms of teaching and learning (European Union, 2018).

Furthermore, preschool children present some developing characteristics that can also be seen as an opportunity: the cerebral structures still in formation, especially in the pre-frontal cortex which is the brain area where important mechanisms happen (e.g., focal attention, awareness, selection, planning; Posner and Rothbart, 1998). Research has indicated that the first signs of self-regulation and metacognition are present in the early stages of life and that it should be potentiated (Bronson, 2000; Bryce and Whitebread, 2012; Whitebread et al., 2005). Although the controversy on how preschool children develop SRL competences, the research supports that, at this age, children become more aware of their thoughts and actions (Bronson, 2000; Pramling, 1986, 1988; Whitebread et al., 2009). Some processes such as organizing, reflecting, modifying the environment, making intentional decisions and solving problems are also more frequent, especially when these skills are often trained (Bryce and Whitebread, 2012; Piscalho and Veiga Simão, 2014). Children use audible instructions to help them organize their work and regulate their behavior, but still have difficulty explaining the content of their thoughts (Alarcón-Rubio et al., 2014; Winsler et al., 2009). At this age, there is an effective lack of vocabulary, because the language management is still in acquisition. Another characteristic of preschool age is that they tend to focus on the action, reflecting in a retrospective way and, therefore, disregarding the need to plan (Sáiz et al., 2014). Educational practices promoting self-questioning enable children to master and internalize the learning process and, consequently, their metacognitive reasoning (Bryce and Whitebread, 2012).

In the SRL process, new learning experiences are constantly enriched by previous ones, even when learners face difficulties. Somehow, it is not the difficulty that drives the learner to learn, but rather the chance to reflect and find ways to overcome it (Grigorenko and Sternberg, 1998). The approaches where an adult stimulates the child's learning potential may help understand whether preschool children face difficulties in the SRL phases and, if so, what difficulties emerge in each phase. Accordingly, it is imperative to reach these objectives with validated assessment methods. Thus, the focus of the present study is to reach these objectives and provide future research with comprehensible and valid resources founded on best practices to measure SRL in preschool children.

### 1.2 Measuring self-regulated learning in preschool

Since self-regulated action is intentional, planned, temporary and dynamic, it is a very complex process to assess (Lopes da Silva et al., 2004). Consequently, its dimensions cannot be assessed separately, as each one does not explain the holistic and multidimensional process. The SRL dimensions, working as a whole, reflect the complexity and the diversity of the learning process as it dependents and is influenced by the situation and the context. For instance, parenting SRL behaviors (e.g., Pino-Pasternak and Whitebread, 2010), the family structure (e.g., Luo and Gao, 2022) and the access to technology (e.g., Araka et al., 2020; Tsiakas et al., 2020) have been studied as relevant aspects that influence the development of SRL in children. Also, research has been trying to understand how different SRL dimensions and processes are developed in a specific system, underlying the importance to contextualize the assessment approach (Kuvalja et al., 2014). Some authors (e.g., Cleary and Callan, 2018) advocate a clearer differentiation between SRL as an “aptitude” and “event”, which may better explain the type of instrument applied to the research. For instance, the instruments that measure SRL as an aptitude, such as self-report questionnaires or teachers' judgements, tend to describe some relatively stable qualities or learners’ attributions, which make it possible to predict their behavior (cognition and motivation). On the other hand, the instruments that measure SRL as an event or activity are characterized as being very complex measures, collecting information on the states and processes applied by learners during their regulation. The think-aloud measures, the methods to detect task errors, the observation methods while the task is being performed, and the diaries are included in this line of research (Winne and Perry, 2000). Recently, attempts to build unbiased instruments to assess SRL as a complex and dynamic process have increased, sustaining that it should be assessed while it is occurring (Boekaerts and Corno, 2005), and through multi-measure approaches (Panadero et al., 2016).

Interviews have been considered a valid instrument to understand the interviewee's experiences and to capture the interaction between the person and the context (De Groot, 2002; Silva and Veiga Simão, 2016). The questions posed to the interviewees should encourage them to reflect on the management of strategies, thoughts, and emotions experienced in the moment. The micro-analytic method made through interviews is consistent to assess SRL since the facilitator (i.e., individual who applies the assessment) has the opportunity to monitor the process in three phases: before, during, and after the interviewee solve the task (Zimmerman, 2013). For each phase, different measures can be applied contributing to an integrative assessment, such as: in the forethought phase, questioning and observation; in the performance phase, think-aloud measures, observation and task solving and, in the self-reflection phase, questioning, observation and stimulated recall (Marulis and Nelson, 2021; Winne and Perry, 2000).

Particularly, the task interview is an instrument that can capture cognitive, metacognitive, motivational, and volitional dimensions of the interviewee's self-regulation in a semi-structured interaction. This instrument is an opportunity to enhance the interviewee's self-knowledge because the assessment approach emphasizes, not only the result of the task, but the processes mobilized to solve it towards a certain learning goal (Marulis and Nelson, 2021; Romera, 2003). The facilitator of the task interview can act as a supporter for growing metacognitive awareness, guiding the interviewee to self-regulate during the execution of a task, especially when the learner is not familiar with reflection practices, such as young children (Marulis et al., 2016). Moreover, the assessment procedure should be strategic to make covert processes into overt planning, performing, monitoring, assessing, and reflecting (Costa, 2014; Romera, 2003). Interviewees' perceptions of task difficulty and interest, as well as self-efficacy beliefs towards the goal can also be captured using this measurement procedure (Bandura, 1997; Silva and Veiga Simão, 2016; Zimmerman, 2013). The interview questions are imperative to lead interviewees to think about how and why a task should be performed, enabling a deeper consciousness of the psychological processes involved in learning (Marulis et al., 2016). This fact is even more important with young children because they do not master language skills and the questioning is an opportunity to increase vocabulary concerning SRL processes and strategies (Robson, 2016). Therefore, when choosing a measure to assess SRL, the instrument's potential and the learners' developmental specificities should be taken into account to ensure that a deeper understanding of the overt and covert processes is gathered.

Other commonly used instruments to assess SRL in young children are the observation measures (Dörrenbächer and Perels, 2018; Howard et al., 2019; Nader-Grosbois et al., 2008; Whitebread et al., 2009). These present some advantages because they do not rely on children's self-report, they provide objective information as a result of external assessment and they have applicability to preschool practices. However, the data triangulation with quantitative and qualitative sources is frequently suggested (De la Fuente and Diaz, 2010; Dörrenbächer and Perels, 2018; León-Ron et al., 2020; Whitebread et al., 2009). Research using observation instruments has also suggested using various types of tasks to activate different SRL dimensions in real-time assessments (Dörr and Perels, 2020; Nader-Grosbois et al., 2008).

The Head-Toes-Kneed-Shoulders task (HTKS; Ponitz et al., 2008; Ponitz et al., 2009; see detailed description in the instruments section) has been widely used to assess self-regulation in preschool (e.g., Hee et al., 2018; Montroy et al., 2016; Puranik et al., 2019; Solé-Ferrer et al., 2019). However, the development of other sensitive and ecologically valid measures to assess young children's self-regulation still constitutes a gap in research (McClelland and Cameron, 2012; Perry, 2019).

### 1.3 A dynamic approach to making covert self-regulated learning processes in preschool visible

Dynamic assessment or dynamic testing (Sternberg and Grigorenko, 2002) has been considered one of the most effective ways to assess learning processes. On the one hand, the dynamic approach allows to assess processes while they are occurring, in an on-going procedure. On the other hand, a relevant role is given to the facilitator as he/she actively engages in the interview. The interaction between the facilitator and the children offers the latter feedback regarding their performance, aiming to underline their learning potential (Budoff, 1987). This type of approach is not simply a test, but an opportunity to intervene in performance changes and individual differences (Panadero et al., 2016). It offers the facilitator the opportunity to instigate the development of learning processes within specific contexts and tasks, especially, when using age-appropriate and context-specific material (e.g., curriculum-based dynamic tests). The instruments designed to perform dynamic assessment can work as a complement to traditional assessments (Hidri and Roud, 2020), as those tools are reactive and motivate the modifiability of the processes instead of their permanency (Grigorenko and Sternberg, 1998). Fundamentally, as learners’ strengths and weaknesses can be identified, this assessment approach provides an opportunity to understand which dimensions need improvement. Some research has been devoted to the topic (Al-Hroub and Whitebread, 2019; Stevenson et al., 2013) and other studies, although not assuming a demarked dynamic assessment approach, have used similar producers to fairly assess SRL processes (De la Fuente and Lozano, 2011; Romera, 2003) and metacognitive awareness (Marulis et al., 2016).

The aforementioned studies and the respective instruments presented seem to highlight how self-regulation is multidimensional (e.g., cognitive, metacognitive, emotional, social, and motivational). Therefore, in order to measure SRL in preschool children, it is essential to consider the measurement instrument, the context in which it takes place (e.g., classroom) and the method of applying it (e.g., appropriateness of the task and the approach to the participants' stage of development). These studies have contributed greatly to knowledge regarding the measurement of SRL in preschool children as they allow for the drafting of the advantages and disadvantages of the various approaches (Appendix 1). However, the instruments intended to capture only a part of the SRL process (e.g., metacognition in Marulis et al., 2016) and small samples were used, thus reducing the findings’ reliability to adapt to other contexts. The literature on SRL in preschool suggests that instruments of dynamic assessment with high content validity and reliability are scarce (Stevenson et al., 2013). Moreover, the design of valid ecologic measures to use in real preschool contexts is still needed, because the majority of the instruments were designed to be used in a laboratory (McClelland and Cameron, 2012).

In view of the theoretical review and recommendations offered by previous research, this study presents a method which was designed to measure SRL in preschool children considering their developmental characteristics (5 year-old children), a curricular infusion approach, and the multidimensionality of the SRL process. Its design was based on the above-mentioned measures and it was tested in an ecological setting (i.e., classrooms). The Dynamic Assessment of Self-regulated learning in Preschool (DASP) method assesses diverse data through a set of measures (e.g., interview, observation, product of the task), to capture learners’ SRL competencies while solving authentic preschool tasks, in a given context and time, and as reliably as possible. By developing and validating this instrument, we propose to understand whether an ecological, dynamic and multidimensional approach can assess both overt and covert SRL processes in preschool children.

We advocate that the novelty presented by this new instrument consists in filling a gap in the literature to design ecologic measures to assess self-regulated learning in preschool, considering it as a dynamic and multidimensional process. The fact that cross-validation data is collected with the DASP method (i.e., observational, verbal, and performance data) while children engage in authentic tasks, it allows to fully evaluate the strategies and processes applied in all the cyclical phases of the self-regulated learning, which is also the framework upon which the instrument was developed. Moreover, the fact that a dynamic assessment approach is considered within the DASP method, makes its usefulness and applicability go further for other assessment measures, helping to foster preschoolers’ competencies that still in acquisition.

In line with the theoretical framework presented and the study's objectives, two research questions were addressed in this study:

RQ 1 - Will preschool children face difficulties in the different self-regulated learning phases? And, if so, what difficulties emerge in each phase?

RQ 2 - Does an ecological, dynamic and multidimensional approach (the DASP method) assess overt and covert self-regulated learning processes and strategies in preschool children?

## 2 Method

### 2.1 Participants

A convenience sample of 214 children that spoke Portuguese or Portuguese-Brazilian fluently was used in this study. Children were between 5 years and 3 months and 7 years and 6 months old (M_age_ = 5.6; SD = .5) and 48% were female. The participants were attending preschool in the Metropolitan Area of Lisbon, Portugal: three schools were private and one school was public. Children belonged to 15 classes that had between 20 and 26 children per class. The participants were from middle-class families and all lived with their mother, or father, or both. Some parents (N = 177) answered and returned characterization questionnaires about topics which may have an impact on children's SRL, such as: access to technology, siblings, parents' qualifications, distance between home and school and daily time to fall asleep and to wake up (Table 1).

**Table 1:** Sample characterization.

Topics		
Technology	Children with access	93%
	Playful purposes	49%
	Learning purposes	5%
	Both	46%
Siblings	Participants having brothers/sisters	76%
	Participants having an older brother/sister	53%
Parents' qualifications	Mother having an academic education	65%
	Father having an academic education	43%
Distance home – school (average)		9 min
Hours' sleep (average)		10h:20m
Fall asleep hour (more frequent)		9:30 pm
Wake up hour (more frequent)		8 am

### 2.2 Instruments and resources

#### 2.2.1 The Dynamic Assessment of Self-regulated learning in preschool (DASP) method

The method incorporates features from other assessment resources (Costa, 2014; Marulis et al., 2016; Nader-Grosbois et al., 2008; Romera, 2003; Silva and Veiga Simão, 2016; Whitebread et al., 2009), including a game-like format and a protocol of task interview adapted to preschool. The design and applicability of the interview recreate a preschool learning situation. The protocol has a paper-pencil format and allows facilitators to take notes on the children's answers in a precise, systematic manner, closely resembling a preschool daily routine. Thus, video or audio recordings were not included in this study. The instrument consists of three parts and its reliability was considered suitable (α = .80 in the forethought phase, α = .52 in the performance phase and α = .98 in the self-reflection phase). More specifically, in the forethought phase there are six questions, and in the self-reflection phase there are eight questions. In both phases the main data collection method is indirect observation through self-report. Atypical events, relevant non-verbal data and some of the children's verbalizations are also registered by the facilitator. In these two SRL phases, the type of interaction between the parties is similar: as the facilitator poses questions, the child endeavors to answer them. For a systematic analysis of the data, the children's answers are registered according to previous categories corresponding to different types of strategies in an ascending order of complexity (see Appendix 2: the DASP method protocol). The children's responses to the questions “1. What do you think you need to do?” (goal identification); “2. Now, complete the sentence: I am going to need…” (organizing and transforming) and “3. How are you going to do the activity?” (establishment of performance goals) of the forethought phase, and the questions “1. Can you explain to me how you did it?” (descriptive assessment); “2. Why did you do it this way?” (strategic approach assessment); “4. Why?” (causal attribution) and “8. Next time, how are you going to do this activity?” (adaptive/defense inferences) of the self-reflection phase, are typified according to their content with a 1, 2, 3 and 4 code. Additionally, the forethought phase questions “4. Do you think you can do this activity even if you are not familiar with it?” (self-efficacy perception); “5. Do you think it is going to be interesting, funny?” (interest of the task perception); “6. Do you think it is going to be easy to do this activity?” (perception of the task's difficulty), and the self-reflection phase questions “3. Did you do it well?” (efficacy assessment); “5. Was it easy to do this activity?” (perception of the task's difficulty); “6. Did you enjoy the activity?” (affective reaction) and “7. Are you happy with what you did?” (self-satisfaction) are answered in a smile format where the children point to their answer, such as, yes ☺, more or less · or no ☹, coded with 1, 2 and 3. In the performance phase, two types of data are collected: the children's strategies/behavior (observation data) and the product of the task, in a more open approach. While children solve the task, the facilitator does not question them and notes the observed strategies in four items: attention focus, self-instruction, resource management and monitoring, and social assistance; the observations are typified according to their presence or absence with a 1, 2 and 3 code. The product of the task is individually analyzed at a post-data collection point in time, applying assessment criteria, and decoding the results (detailed description in the next section). The interview start and end time is also recorded in the protocol for characterization purposes.

**Figure 1:**
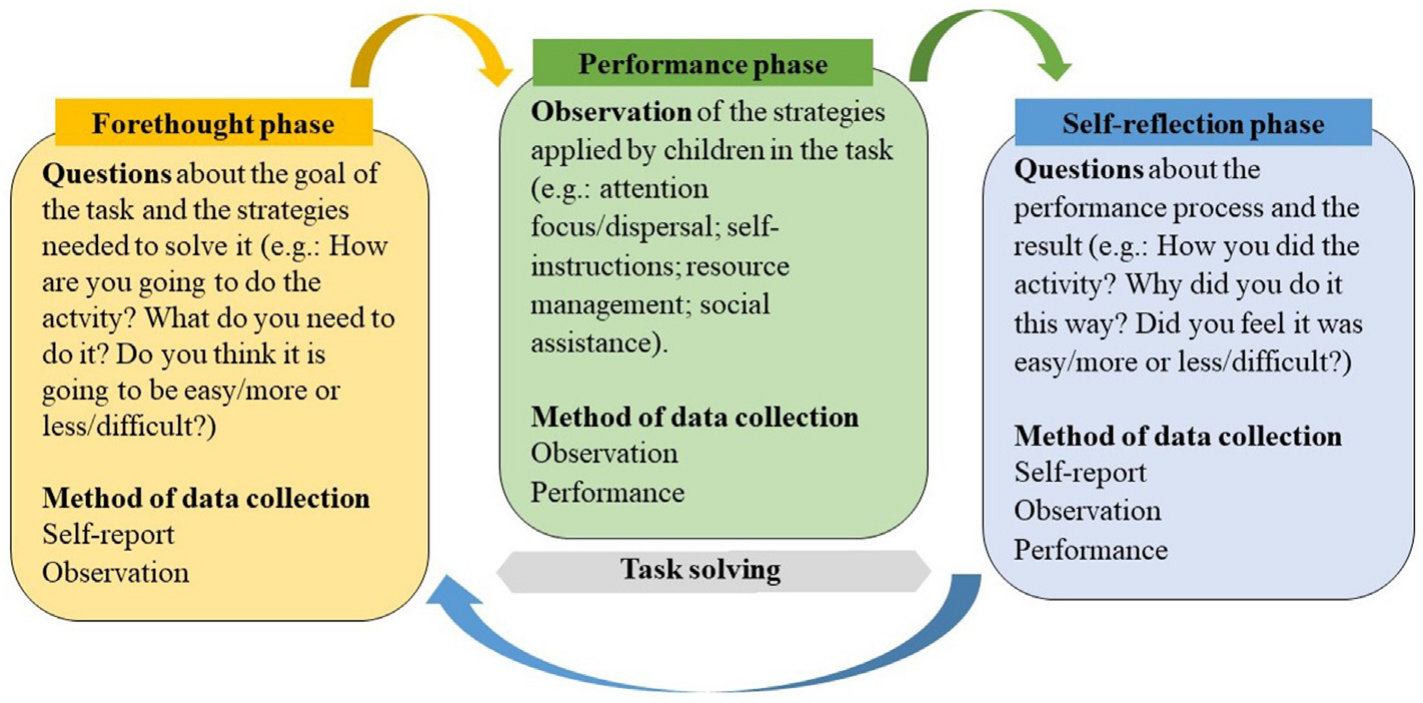
Multimethod approach regarding the SRL model.

This type of procedure to assess SRL competencies in preschool and the category system was developed on the basis of other instruments (e.g., Costa, 2014; Marulis et al., 2016; Nader-Grosbois et al., 2008; Romera, 2003; Silva and Veiga Simão, 2016; Whitebread et al., 2009). The categories were selected according to the SRL model (Zimmerman, 2013), and judiciously discussed and analyzed by experts in the SRL field. Additionally, a pilot study was conducted with preschoolers to pretest the instrument (Silva Moreira and Veiga Simão, 2020). This approach embraces both the intervention and the assessment perspective (Panadero et al., 2016). The content validity of the method was assured by its solid theoretical model and the protocol questions were in line with the theory (Zimmerman, 2013). It was also designed to respect the recursive dynamism of the application procedure. The criterion validity was reinforced by the reactivity effect of the method (Panadero et al., 2016). Therefore, different types of measures were used in each phase seeking to capture SRL processes from a dynamic, cyclical, and multidimensional perspective. The specific measures considered the developmental characteristics of the children (Ferreira et al., 2015; Lopes da Silva et al., 2004), such as their difficulty to speak about self-regulatory processes. Self-reported data was complemented by other sources, namely observation data and the product of the task, with a view to data triangulation. Accordingly, the multimethod data collection of the DASP method aimed to assess the complexity of the SRL strategies and processes in a dynamic manner (Figure 1).

One preschool task was selected to be solved by children during the interview, considering specific developmental and curriculum aspects. This task was authentic because the research option was a curricular infusion model, where children could feel the situation as resembling their everyday life. To assess self-regulation in preschool with the DASP method, any task can be used according to the following principles:

a) the task should allow to mobilize SRL strategies and processes being applied in the specific context and while it is happening (Boekaerts and Corno, 2005);
b) it should enable children to plan, monitor and assess performance according to the tasks' initial goals;
c) the task should include curricular content mastered by children (e.g., 5 years old skills), so the facilitator can ensure that children fully exercise previous knowledge and the assessment results are referring to SRL competency development and not to the unfamiliarity with the content of the task;
d) the type and the number of cognitive operations should be according to the curriculum, the children's developmental stage and the standard educational practices for this age, as the task should be accessible to resolve, but sufficiently challenging to potentiate SRL competencies (e.g., solving problems).

The task used in this investigation was the “Clown task”, a cognitive task that had been previously piloted and found to be appropriate for preschool children (Costa, 2014; Silva Moreira and Veiga Simão, 2020) according to educational guidelines. It included content about geometrical figures (recognizing and replying), and numeric notions (count from one to three). The task presented individually to children had two clowns: a model on the left side of the page with three triangles, three circles, and two squares drawn as clothing patterns, and, on the right side of the page, there was a similar clown but its clothes had no patterns. The task instruction was: "Draw the same number of triangles, circles, and squares in the clown on the right side.” To solve the activity, some material was available on the top of the table (e.g., pencil, pen, color pencils, eraser, etc.). Children took 3–5 min to solve the task.

#### 2.2.2 Head-Toes-Knees-Shoulders (HTKS)

To perform concurrent validity for the Clown task (Murphy and Davidshofer, 1998), the HTKS behavioral task (Ponitz et al., 2008, 2009) was applied using the same DASP method. This task has been used to assess behavioral and motor regulation in preschool children. According to the authors, the HTKS allows the assessment of behavioral regulation, including inhibitory control, attention, and working memory. Attention processes include focusing, sustaining and shifting attention; working memory includes holding information while processing new information, and inhibitory control is observed by stopping an automatic response deliberately to exhibit another behavior. The instrument's reliability was considered suitable (α = .85). During the task application, the facilitator asks the child to first touch his/her head and then touch his/her toes. Then, he/she is asked to do the opposite and touch his/her head instead of his/her toes. The task includes three parts involving paired rules: a head-toes section, a knees-shoulders section, and a section with four types of paired commands. There is a total of 20 items in each part. Children's behavioral answers are registered on a paper protocol. Children need to score, at least, four points to skip to the next part. It requires 5–7 min to be administered.

### 2.3 Procedures

Ethical issues were assured and the research was approved by the Commission of Deontology of the Faculty of Psychology of the University of Lisbon. Anonymous participation was guaranteed through a code of identification. The facilitator was a researcher with a Master degree in Education Psychology, developing a PhD project, with vast experience in research projects with children, and who was actively engaged in all the procedures, including the collection of the school management and parents' consent. Before the individual task interviews with children, some external effects were minimized by daily visits to preschool activities. In other words, the interviewer spent some time in the preschool classrooms aiming to establish a relationship of trust with the children and to become a familiar face in the daily activities (Amado and Ferreira, 2013; Brenner, 2006). The interviews took place in a quiet room or hallway next to the participants' classroom where only the facilitator and the child were present. The place had two chairs and the material to perform the tasks was placed on a table. The same procedure was repeated with each participant and interruptions were avoided. The facilitator started by appreciating the children's participation, letting them observe the task, and explaining the instructions. The first part of the interview was the application of the DASP method with the Clown task. Sequentially the method was applied with the HTKS task. In the end of the interview the facilitator remained available for children's questions and then dismissed them, thanking for the collaboration. The interview procedure was conducted by a single facilitator who completed the protocol checklists to assure standardization; the accuracy of the checklists were then rated by two expert raters. Interviews varied from 10 to 40 min. Parents' questionnaires were filled at home or in the school's parents meetings.

### 2.4 Data analysis

A distinct type of statistical analysis from the Classical Test Theory was selected as we considered that the Item Response Theory (IRT) would allow us to better understand children's ratings. This type of analysis establishes a link between the properties of the items, the participants' responses and the trait being measured. We found this analysis to be reliable and conceptually powerful (Embretson and Reise, 2000) and it could give some good indicators for the items' adequacy for preschool children. Some previous studies had already applied this analysis (Camilli and Kim, 2018; Ferreira et al., 2015; Francisco et al., 2015; Stevenson et al., 2013; Tibi et al., 2021) and its innovative approach seams promising. Specifically, we proceeded with the Rasch analysis (Rasch, 1980) with the *Winsteps* program (Linacre, 2019) to study the internal consistency of the instrument's items, how the task fit participants and how children perceived the difficulty level of the task. In the simplest IRT model, the chance that an item is solved correctly depends on the difference between the latent skill of the respondent and the difficulty of the item. The change score has the same meaning across the whole range of the measurement scale about the probability of obtaining a correct *vs.* incorrect answer, which enables practitioners to rely on the instrument's reliability. The IRT analysis made it possible to calibrate both the participants and the instruments' performance (e.g., difficulty level) on a common scale (De-Mars, 2010; Embretson, 1996). This statistical procedure allowed us to interpret the accuracy or difficulty with which the children performed the tasks and to ascertain whether the instrument itself was valid for this purpose. This statistical procedure also enabled us to verify the unidimensionality of each self-regulation phase, as well as to measure how both the children and the task performed in interaction with each other in a specific situation (DeMars, 2010; Embretson, 1996). This measurement procedure provided an analysis of the interactions between the children and the tasks, which aided the interpretation of the variables to be measured.

In the present study, all items were analyzed to understand whether they fit the model (p < .01) or whether there were items with excessive infit and outfit mean square residuals. That is, we considered removing infit standardized mean squares higher than 1.4 and outfit standardized mean-squares higher than 2.0, as suggested in the literature. Good reliability levels were considered above .70 (Bond and Fox, 2007). Thus, model 1 was run with all the participants and, as the reliability levels were low, a second analysis, commonly referred to as model 2, was run excluding the participants presenting infit or outfit values, so the best model could be reached where reliability levels were satisfactory.

Children's responses in the DASP method questioning were processed from qualitative to quantitative data. The answers were categorized from 1 to 4 where a higher score (4) indicate a more complex performance of strategy use, the middle values indicate common strategies and the lower score (1) signify an irrelevant response or a non-response. The strategies applied by children during the performance phase were explored through frequency analysis. Some of the qualitative observations and children's verbalizations collected during the assessment time were analyzed through content analysis helping quantitative results to make sense in terms of interpretation.

The Clown task was analyzed as performance data. We processed the qualitative data quantitatively to analyze the goal task achievement (e.g., 1 – goal task achieved; 2 – goal task not achieved). Furthermore, a content analysis of 214 task products was performed. The competencies required by the task were categorized (e.g., graphic competencies, numeric notions, and spatial orientation competencies) and its frequency analysis was organized in three levels (e.g., incorrect, partially correct, and totally correct). In fact, the criteria to analyze the product of the task was adequate to the tasks’ specificities and the competencies expected to be evaluated. The Clown task was assessed in a system of mutually exclusive categorization by two judges with an intraclass correlation of 98%.

In the HTKS task children's behavioral answers corresponded to: incorrect–0 points; self-corrected–1 point; correct–2 points. The lowest score was 0 points and the highest was 60 points. Higher scores should indicate higher levels of behavioral regulation. The participants' performance was analyzed through the sum of the correct answers given in each of the three parts of the task.

## 3 Results

Upon close examination of the results of each SRL phase, the processes and strategies applied by the children, and the product resulting from the activities performed by the latter, this section presents the findings in light of the research questions: RQ 1 - Will preschool children face difficulties in the different self-regulated learning phases? And, if so, what difficulties emerge in each phase? RQ 2 - Does an ecological, dynamic and multidimensional approach (the DASP method) assess overt and covert self-regulated learning processes and strategies in preschool children?

The IRT analysis enabled us to confirm the suitability of the method for preschoolers and their level of difficulty (RQ 1). Each SRL phase was analyzed and the forethought and self-reflection phases are explained first, instead of following the chronological order of the method (i.e., forethought, performance and self-reflection phase), due to the similarity of the procedure in both phases: the facilitator posed questions to which the children responded verbally (indirect observation). The results of the performance phase are then presented, taking the different type of data concerning the other two phases (direct observation) into consideration; at that point, the children solved the task independently and the facilitator did not intervene (for a detailed description, see the method section). Finally, the IRT analysis for the two tasks performed by the children are shown.

**Figure 2:**
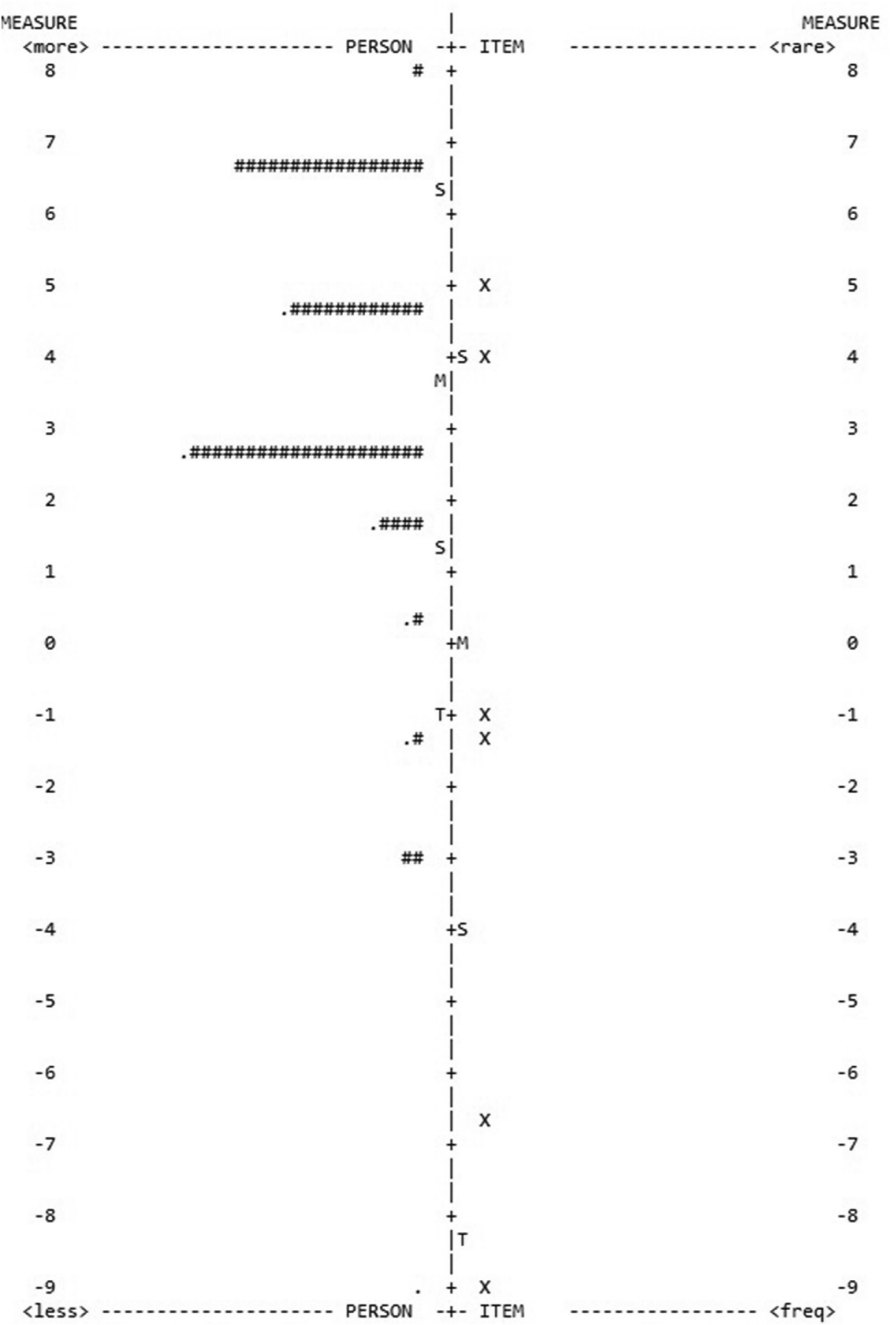
IRT map for the best model in the forethought phase regarding the Clown task.

According to the IRT analysis in the forethought phase, model 1 pointed to low reliability for both tasks (Table 2) and several participants revealed outfit (39 participants with outfit in the Clown task and 83 participants with outfit in the HTKS task). The best model (model 2) dismissed the participants' outfit, showing the highest scores for both tasks in person fit (.42–.75 in the Clown task; .54 to .84 in the HTKS task). Although the reliability results yielded an ascending score (.35–.75 in the Clown task; .46 to .58 in the HTKS task), they did not attain the optimal level. The IRT map captured the respondents’ distribution (Figure 2), highlighting the suitability of the forethought phase for preschoolers as it suggests similar levels of difficulty (i.e., the observations are almost all placed in the same quadrant of the graph).

**Table 2:** IRT models regarding the forethought phase.

SRL phase		Clown task		HTKS task	
		Person fit	Reliability	Person fit	Reliability
Forethought	Model 1	.42	.35	.54	.46
	Model 2	.75	.65	.84	.58

**Figure 3:**
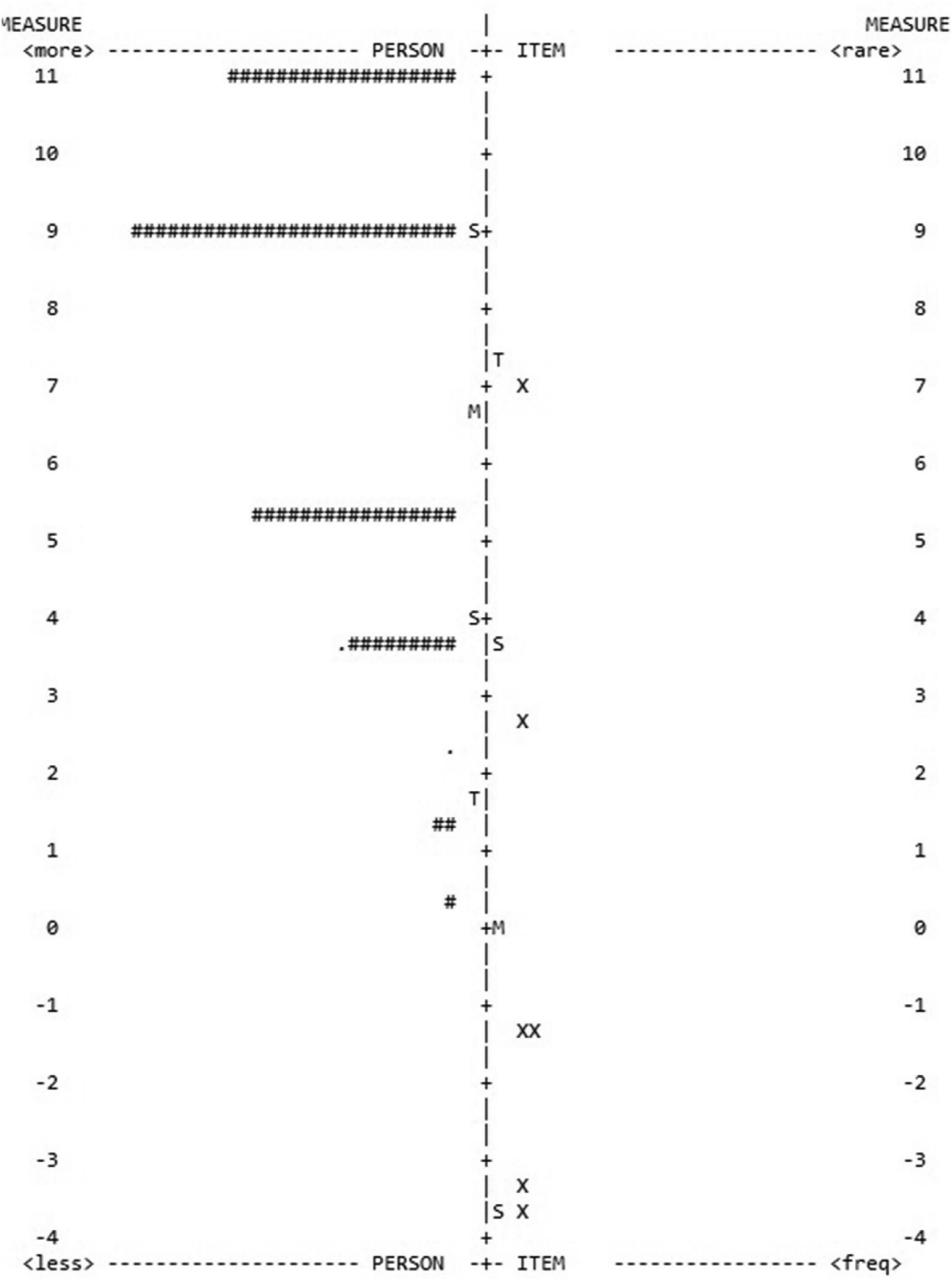
IRT map for the best model in the self-reflection phase regarding the Clown task.

The self-reflection phase results of the IRT model 1 showed low reliability for both tasks (Table 3) and many participants were outfit (44 participants with outfit in the Clown task and 98 participants with outfit in the HTKS task). The outfit participants were then dismissed and the best model showed a significant increase in person fit for both tasks (.29–.63 in the Clown task; .53 to .84 in the HTKS task). Despite the reliability score increase in model 2 for both tasks, it did not reach an optimal level. The IRT results on the self-reflection phase also showed that, for both tasks, the children struggled to answer the 8^th^ item (*How are you going to do this activity the next time?*). Other items revealing some outfit scores in the HTKS task were those related to the strategic approach assessment (*Why did you do it this way?*), and the item regarding efficacy assessment (*Did you do it well?*). The IRT map illustrates the respondents’ distribution (Figure 3). In other words, most of the participants found the self-reflection phase difficult, although it was performed easily by some outliers (i.e., observations placed next to 0 and 1).

**Table 3:** IRT models regarding the self-reflection phase.

SRL phases		Clown task		HTKS task	
		Person fit	Reliability	Person fit	Reliability
Self-reflection	Model 1	.29	.32	.53	.52
	Model 2	.63	.60	.84	.64

Results concerning the IRT analysis on the performance phase yielded the lowest scores when comparing the three SRL phases. As the instrument presented high content validity in theoretical terms (see performance phase scores in Table 4), the results suggest that the children found this SRL phase difficult. Even when the outfit participants of the first model were dismissed (29 in the Clown task and 25 in the HTKS task), the person fit (.00 in both tasks in the first model) and the reliability level did not increase significantly (.18 in the Clown task and .21 in the HTKS task in model 2 - Table 4).

**Table 4:** IRT models regarding the performance phase.

SRL phase		Clown task		HTKS task	
		Person fit	Reliability	Person fit	Reliability
Performance	Model 1	.04	.00	.00	.00
	Model 2	.00	.18	.00	.21

**Figure 4:**
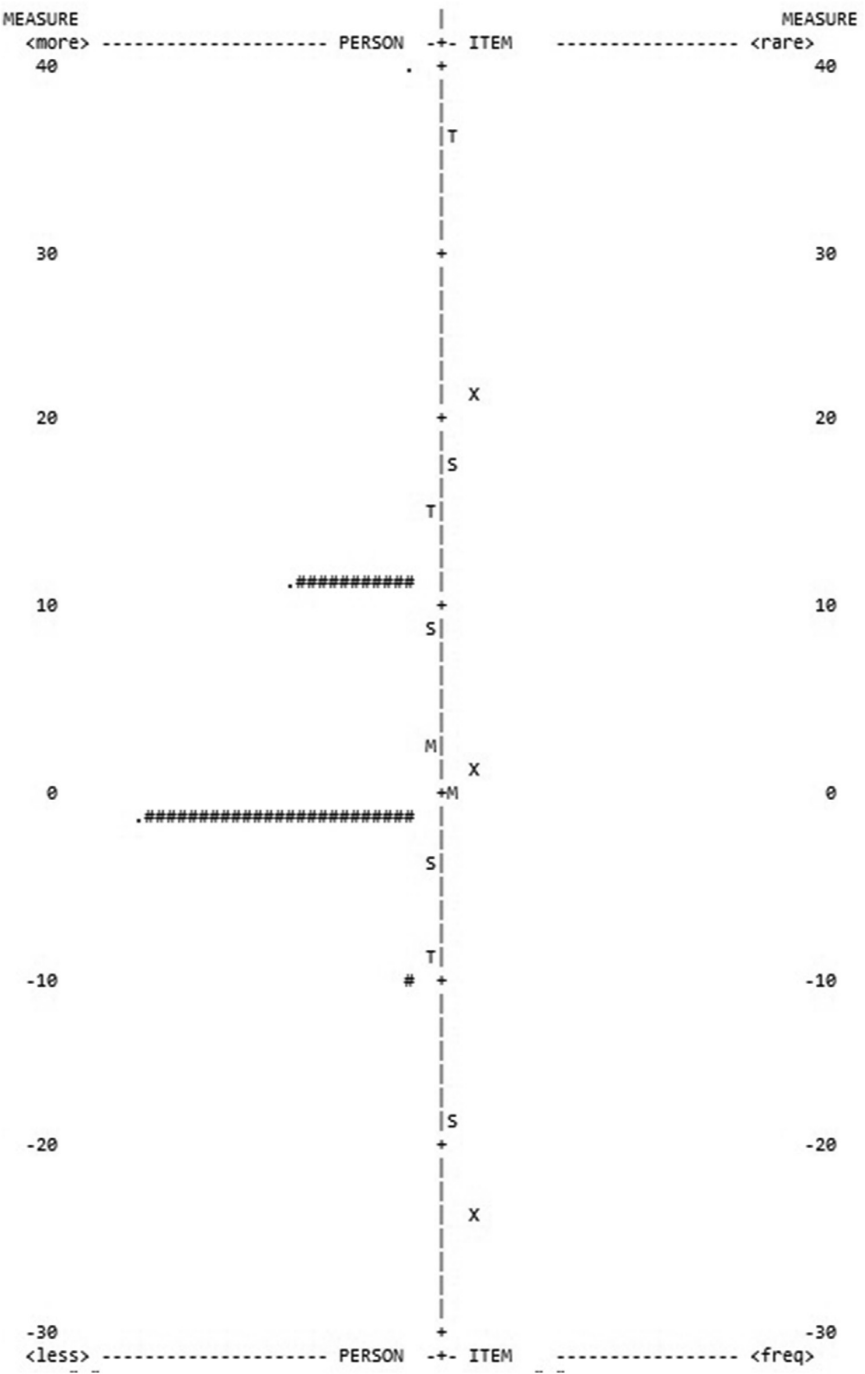
IRT person-item map for the Clown task performance phase.

The performance phase data is confirmed by the person-item distribution in the Clown task where the participants are placed together, suggesting that the requirements of this SRL phase were suitable for preschoolers (Figure 4).

To run the IRT analysis for the Clown task and the HTKS task, all the participants and items were included in model 1. Comparatively, the Clown task scored lower in person fit (.66) than the HTKS task (.77), showing that children had more difficulty responding to the former. The item fit showed that both tasks were suited to 5 year-old children, who scored .97 in the Clown task and 1.00 in the HTKS task. Considering the reliability score of model 1, a second analysis was run without the outfit participants (30 in the Clown task and 34 in the HTKS task), and higher results were presented (see model 2 in Table 5). The scores displayed good internal consistency of the items, namely .92 in the Clown task and .85 in the HTKS task, pointing to the tasks' suitability for the participants.

**Table 5:** IRT models regarding the tasks.

Tasks	Clown task			HTKS task		
	Person fit	Item fit	Reliability	Person fit	Item fit	Reliability
Model 1	.66	.97	.84	.77	1.00	.75
Model 2	.89	.97	.92	.98	1.00	.85

Seeking to answer the second research question, i.e.: RQ 2 - Does an ecological, dynamic and multidimensional approach (the DASP method) assess overt and covert self-regulated learning processes and strategies in preschool children? the IRT approach enabled us to study the suitability of the DASP method for the participants in terms of the SRL questions. The item fit results showed that this assessment method was reliable from a conceptual point of view (Table 6).

**Table 6:** Item fit regarding the three SRL phases.

SRL phases	Clown task	HTKS task
	Item fit	Item fit
Forethought	.99	.99
Performance	1.00	1.00
Self-reflection	.99	.99

As with the first research question, we chose to initially present information on the forethought and self-reflection phases and then on the performance phase due to the type of data collected in each SRL phase. Thus, with the content analysis run from the systematic annotation, it was possible to triangulate the concurrent data in order to gain a deeper understanding of the DASP method's potentialities. More specifically, the forethought phase categories were crossed with the task's goal achievement in the Clown task to, on the one hand, complete the self-report data with observational notes and, on the other, test associations between the data that could contribute to stronger and more interesting results. For instance, more than half of the sample was able to anticipate some of the resources needed to perform the task (e.g., “I need a pencil.”; “I need a sheet with a clown.”), but they did not achieve the goal. The percentage of children anticipating strategies (e.g., “I need to count.”; “I need to pay attention to do the activity.”) or resources and strategies (e.g., “I need a pen and to be focused.”) was not above 6%. With a small difference of 2%, the number of children able to anticipate the action, describing it in detail was higher (e.g., "First, I am going to count the figures, then draw the triangles, after the circles and finally the squares.") than those who pointed or referred to the task's goal non-verbally (20%–18%, respectively). In this category, overestimation of the children's perception was evident, as the higher percentages belonged to the children who did not achieve the task's goal (Table 7).

**Table 7:** Relationship between the achievement of the goal in the Clown task and some of the strategies applied by the children in the forethought phase.

Forethought phase					
Category	Organizing and transforming			Establishment of performance goals	
Response item	Anticipates resources	Anticipates strategies	Anticipates resources and strategies	Points or refers to the task goal non-verbally	Anticipates the action in detail
Goal task achieved.	24%	6%	1%	7%	10%
Goal task not achieved.	54%	5%	3%	18%	20%

The frequency analysis strategically detached from the self-reflection phase categories (Table 8) highlighted that the children's responses reflected a growing metacognitive awareness. In fact, some of the children were able to describe details from the performance phase retrospectively. They explained their choices and referred to personal skills to justify the task outcome (e.g., “I did it like this to be the same as the model.”; “Because I wanted to do it the way I knew how to/the easiest way.”). These can be particularly interesting signs of children's intentionality and a developing awareness of their agency in the learning process. The most complex adaptive or defense inference (emphasized through the question *How are you going to do this activity next time?*) included the use of cognitive or metacognitive strategies to improve the task outcome. Although this was the most difficult question for the children, as previously shown in the IRT results, the fact that some data can be presented may be indicative of a developing abstraction skill in our sample of preschoolers.

**Table 8:** Relationship between the achievement of the goal in the Clown task and some of the strategies applied by the children in the self-reflection phase.

Category	Self-reflection phase		
	Self-judgement – Descriptive assessment	Self-judgement –Causal attribution	Self-reaction – Adaptive/defense inferences
Response item	Makes the descriptive assessment of the task in detail	Attributes internal causes to the result	Names cognitive or metacognitive strategies to improve the result
Goal task achieved.	19%	23%	27%
Goal task not achieved.	44%	59%	51%

The self-efficacy and difficulty perceptions in relation to the Clown task suggested a tendency for children to overrate their answers, claiming to feel sufficiently skilled to solve the task while the majority in fact, failed to accomplish the task's goal. The results regarding the difficulty of the task are in line with the IRT results for the item fit showing that the children found the task easy. Relying on children's self-report, they assessed the task as easy prior to and following the performance phase, with a small number of participants changing their perception in a positive manner (Table 9).

**Table 9:** Relationship between the achievement of the goal of the Clown task, self-efficacy perception and perception of the task's difficulty in the forethought phase, and self-judgment on efficacy and self-judgment on the difficulty of the task, in the self-reflection phase.

	Forethought phase						Self-reflection phase					
	Self-efficacy perception			Perception of the task's difficulty			Self-judgment on efficacy			Self-judgment on the difficulty of the task		
	☹ unable		☺ able	☹ difficult		☺ easy	☹ unable		☺ able	☹ difficult		☺ easy
Goal task achieved.	2%	2%	29%	5%	5%	24%	0%	7%	27%	3%	3%	26%
Goal task not achieved.	1%	5%	60%	5%	11%	51%	0%	6%	60%	3%	6%	57%

The content analysis of the performance phase also allowed for the identification of several strategies used by the children (Table 10), where the most common strategy was the management of resources towards the goal (98%), and the less used was the request for social assistance – only 3% of the participants asked for help.

**Table 10:** Observable strategies and corresponding percentage of participants that applied them while performing the Clown task.

Performance phase		
Category	Strategies	
Attention focus	Interruption	12%
	Maintenance	88%
Self-instruction	Uses audible self-speech	37%
Resources management and monitoring	Manages resources towards the goal	98%
Social assistance	Asks for help	3%

**Figure 5:**
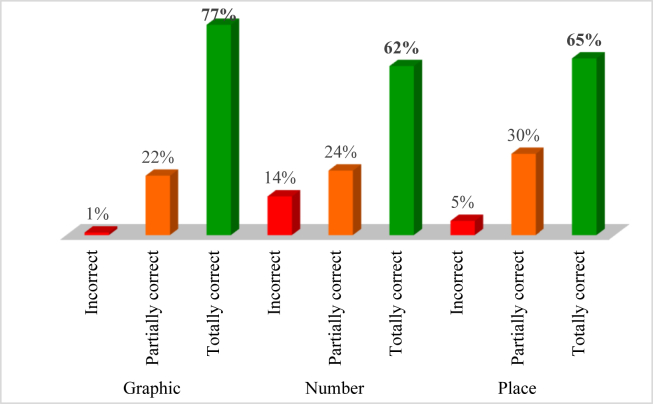
Results regarding the assessment of the Clown task product; criteria included graphic competencies, numeric notions, and placement of the geometrical figures.

The task products' analysis complemented the results regarding the process of task self-regulation yielded in the forethought, performance and self-reflection phases. In other words, the content analysis of the Clown task showed that 34% of the participants achieved the task's goal. To completely achieve the goal, the children had to draw all the geometrical figures – three triangles, three circles, and two squares – following the model on the left side of the page, drawing in the empty outfit of the clown on the right side of the page. Although few of the participants achieved the goal, this result was considered uninformative, and therefore a more accurate assessment was conducted to investigate specific strategies applied by the children. The performance criteria allowed us to assess graphic competencies, numeric notions, and the positioning of geometrical figures (Figure 5).

**Figure 6:**
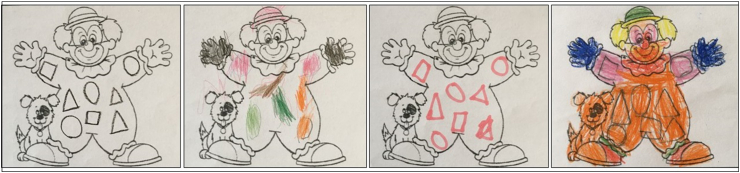
Products of the Clown task.

Considering the three criteria separately, most of the children were able to respond correctly in each one: 77% of the participants drew the 8 geometrical figures adequately while using the competencies expected for 5-year-olds; 65% of them drew the figures in the same place as the model (although this topic was not included in the task's goal), and 62% of the children made the same number of figures that were in the model. Therefore, it appears that analyzing the performance strategies and choices made by children can help to better understand the processes involved in task solving. This content analysis revealed that most of the children mastered the geometrical figures required by the task and displayed no difficulties in recognizing them. Nonetheless, some of them struggled with their replication. Thus, the children's graphic competencies were also analyzed as this skill is closely connected to other competencies and is particularly important in the preschool developmental stage. Some of the psychomotor strategies applied by the children were identified, such as using only one type of resource to draw all the figures (e.g., a black pencil or a blue pen), whereas others used different colors (e.g., blue for the triangles, green for the circles, etc.). Some children expressed an intention to correct their work, using the rubber to erase and draw the pictures again, while some drew the pictures scattered inside the clown, and others drew the forms together without a space between them. Some examples of the Clown task product can be seen in Figure 6.

The HTKS results showed that most children reached the third level scores, with 4 participants achieving the highest score (60 points). A minority of the participants (15) achieved 10 or less points.

Systematizing the information described in the previous paragraphs, some descriptive data concerning the Clown task and the HTKS is reported in Table 11. In addition, a positive and moderate correlation was found between the tasks (*ρ*(209) = .408, *p* < .001).

**Table 11:** Descriptive data concerning the Clown task and the HTKS.

Clown task			HTKS	
Performance criteria	Task goal achieved	34%	Level 3 achieved (40 a 60 points)	86%
Graphic competencies	Totally correct	77%	Level 2 achieved (20 a 40 points)	10%
	Partially correct	22%	Level 1 achieved (0 a 20 points)	3%
	Incorrect	1%	Maximum score achieved	2%
Numeric notions	Totally correct	62%	Minimum score achieved (10 or less points)	7%
	Partially correct	24%		
	Incorrect	14%		
Placement of the geometrical figures	Totally correct	65%		
	Partially correct	30%		
	Incorrect	5%		

## 4 Discussion

Concerning the gaps in the literature on the dynamic measures to assess SRL as a multidimensional process duly adapted to preschoolers' characteristics, this research sought to validate a method to fill those needs. In this section, the findings are discussed according to the aforementioned research questions. The implications for practice are highlighted, as are the study's limitations, and recommendations for future research are advanced.

As for the first research question, RQ 1 - Will preschool children face difficulties in the different self-regulated learning phases? And, if so, what difficulties emerge in each phase? the results provided answers to this question. In the following paragraphs detailed information revealed by the findings and their intersection with the literature are presented, contributing to an understanding of what and how children reported, performed and felt in each SRL phase. Due to the similarity of the procedure applied in the forethought and the self-reflection phases, they are presented sequentially, instead of following the chronological order of the method; the performance phase specificities are then explained.

### 4.1 Specificities of self-regulated learning in preschool

#### 4.1.1 Forethought phase

Regarding the forethought phase, the results reinforce the literature by showing that, in preschool age, planning strategies are not consciously applied by children before starting to solve a task, and they usually plan in action (Bronson, 2000; Sáiz et al., 2014). In fact, most children started to complete the task once they saw it. Children revealed difficulty in anticipating their actions (i.e., “First I will take a pen, then I will look to this one, after…”) when compared to the anticipation of resources or strategies, which goes in line with the maturating psychological processes at this age, such the abstract competencies (Bennett and Müller, 2010; Bronson, 2000). As expected (Bandura, 1997), the participants tended to overvalue their self-efficacy performance in completing the task: 89% of the participants perceived themselves as being capable to perform the activity. The percentage of children assessing the task as easy was also high (75%). Concerning the authentic preschool task used, the Clown task, children could establish their own performance goals, attributing to the task a personal interpretation by, for instance, choosing between some graphic material (color pen, pencil, eraser, etc.) or deciding where to draw the geometrical figures in the paper. Those examples may lead to a deeper reflection on how preschool children can regulate their learning process (Bryce and Whitebread, 2012; Silva Moreira and Veiga Simão, 2019). Moreover, the questioning approach applied in this study seems to be feasible to replicate in daily educational practices, promoting experiences where children are actively engaged in the learning process. The goal of such opportunities should help them to overcome obstacles and progressively learn to think about thinking – metacognitive awareness (Ferreira et al., 2015; Marulis et al., 2016; Romera, 2003). Analyzing the IRT low-reliability levels, we advocate that those results were not due to the difficulty of the items. Rather, some participants may have a lack of training in SRL phases in their daily activities, specially, regarding planning issues.

#### 4.1.2 Self-reflection phase

In the self-reflection phase, participants reflected about the performance phase, *looking back*, observing, reflecting, and trying to explain the solving process and the outcome of the task. This phase was relevant to identify metacognitive strategies as the use of language and thinking required in the revision process made visible some covert processes applied during the task (Marulis and Nelson, 2021; Pramling, 1986). The facilitator may have acted as a promoter of children's expressions, trying to expand their own systems of self-regulation, uncovering some developing psychological processes through language. Due to the developing brain structures, psychological processes such as working memory, abstraction, and attention still reveal low proficiency (Bennett and Müller, 2010; Fitamen et al., 2019; Posner and Rothbart, 1998), but some children were able to explain in detail how and why the task was solved. The participants found the tasks to be easy and enjoyable (97% of the participants enjoyed doing the Clown task and 95% felt the HTKS to be pleasant), which may have positively influenced the way they engaged in the solving process. Although, children felt the 8^th^ item difficult (*How are you going to do this activity next time?*), and many of them were hesitant and less familiar with the idea of referring any task improvements to the next time. Those adaptive and defensive inferences would certainly require a refined conscience and, as we had seen, that is one of the developing characteristics at 5 years of age. Anyway, some children answered the question, showing a growing competence of abstraction, metacognition, self-criticism, redefinition, and adaptation (e.g., “I am going to do better”; “This part won't be like this”; “I will copy the new picture.”) (Bennett and Müller, 2010; Bronson, 2000; Piscalho and Veiga Simão, 2014). In spite of the difficulty felt about this item, the SRL processes here involved are central (i.e., adaptive/defensive inferences), and we claim for its maintenance concurring to the Zone of Proximal Development (Vygotsky, 1978). That is, the SRL approach should be reachable but sufficiently challenging to potentiate new or less developed competencies, sustaining the notion of a dynamic assessment where children's learning potential is developed – a method with both assessment and intervention elements.

#### 4.1.3 Performance phase

In the performance phase, children experienced more difficulty than in the two other phases. In fact, children found it difficult to verbally expressing the content of their thoughts (Bronson, 2000; Winsler et al., 2009). They tended to get lost in their ideas, even if the task was planned properly. Therefore, some strategies mentioned in the forethought phase were not mobilized to the performance phase, as the two phases tended to be treated as distinct instead of a continuity of each other. One of the most useful strategies in this SRL phase is self-speech where audible instructions are verbalized in a sequence of steps (Alarcón-Rubio et al., 2014; Winsler et al., 2009). It allows one to *write* an external script that gets progressively internal, becoming an essential self-regulation exercise. In this study, a small percentage of the sample (37%) used audible instructions, which may explain their difficulties. However, they solved the task in an autonomous way without any verbal support of the facilitator. Previous research had already documented that, in the SRL field, the monitoring, volition, and motivational control processes are less present in educational practices than the planning and reflection phases (Silva Moreira and Veiga Simão, 2019).

Secondly, we aim to justify the sustainability of RQ 2 - Can an ecological, dynamic and multidimensional approach assess overt and covert self-regulated learning processes and strategies in preschool children? by presenting the potentialities of the DASP method and how it could respond to the research needs in the SRL assessment with presc-hoolers.

### 4.2 Potentialities of the DASP method

The DASP approach showed to be a useful instrument to actively engaged children in learning process, namely through the opportunity to plan, perform and reflect about a specific task. The real-time interaction between the child and the facilitator helps children to train and improve learning competencies commonly applied in daily preschool activities. The high content validity of the method, provided by the strong conceptual framework (Zimmerman, 2013), reinforces its reliability to assess SRL processes and strategies. The fact that the SRL was considered, both in the specificities of each phase and in its cyclic functioning as a whole, may have allowed children to get a higher conscience of the strategies mobilized, thus, contributing to a growing metacognitive awareness (Bryce and Whitebread, 2012; Dörr and Perels, 2020; Perry, 2019). We aimed to validate a measure that is adequate for preschool children, considering the lack of instruments adapted for 5 year-old children (Jacob et al., 2020; McClelland and Cameron, 2012). That goal seemed to be achieved as children felt the method to be manageable and understandable. The developmental specificities of preschool children and the complexity of the theoretical concept led to the design of a multimethod approach where several measures were collected simultaneously (Azevedo, 2020; Erdmann and Hertel, 2019; Jacob et al., 2020; León-Ron et al., 2020); the self-report data was complemented with observation data, and the product of the tasks. Trying to overcome other studies' gaps, this investigation intended to obtain information from the preschool children directly instead of their teachers or parents, questioning children on their SRL behavior (Dörrenbächer and Perels, 2018). The *online* (i.e. real-time) observation applied with the DASP method allowed to assess SRL in preschoolers during their learning process by encouraging children to speak about their thoughts and actions according to think-aloud protocols (Dörr and Perels, 2020). The questioning stimulated the dyad thought-language, helping children to progressively get an internal script for their actions. This practice may help children to develop a conceptual understanding of learning and how it is changing through the solving of the task and, in a broader sense, along preschool activities. That is, when a chance is given to young children to judge and reflect on their learning process, they may get aware of what they already know and what they still need to learn to be successful, developing their metacognitive awareness, which may provide a necessary step for SRL (Jeong and Frye, 2020). For instance, when the forethought questions were posed by the facilitator, children could anticipate what and how they would do afterwards. Identically, the self-reflection phase experienced with an external guidance allowed children to think about their own performance, difficulties, aspects to improve, etc. So, the regulation through questions focused the child to explicitly think about the resources, the competencies and the processes needed to solve the task. Research is pointing that this kind of educational practices, when repeatedly and intentionally practiced, may help to turn the external into internal regulation, promoting children's autonomy in the learning process (Saraç and Tarhan, 2020). The DASP method showed positive signs on the interaction and dialogue between the facilitator and the child because it effectively concurred to uncover SRL processes and strategies. The intervening component of the method led the facilitator to give feedback to the child about his/her performance, trying to bring to light the learning potential (Grigorenko, 2009).

In the present study, we performed concurrent validity with the HTKS, a behavioral self-regulation task that is internationally used to assess self-regulation in preschool. The results suggested that different SRL dimensions can be assessed through the DASP method and even those self-regulation tasks where the motor dimension is stronger (e.g. HTKS) may benefit from a dynamic approach. In this case, the activation of some the SRL dimensions (e.g., cognitive, metacognitive, motivational, emotional; Bronson, 2000; Dörrenbächer and Perels, 2018; Whitebread et al., 2009) was prompted by the tasks but also by the method design, encouraging children to verbalize their perceptions and emotions. As the literature points that less attention has been paid to the emotional side in self-regulation (Perry, 2019), our findings are also contributing to understand how those processes are developing at such a young age.

The analysis run with the IRT instead of the Classical Test Theory allowed to better understand how the instrument fitted to the participants. The identification of the children perception about the items difficulty reveled the type of questions more frequently used in preschool, but also allowed to recognize the dimensions that are probably less emphasized and should be promoted. Some studies involving preschoolers have applied the IRT analysis to study digital resources (e.g., Stevenson et al., 2013) or a specific curricular content like Mathematics (Camilli and Kim, 2018), and Arabic letter knowledge (Tibi et al., 2021) but, as far as we know, none validated an instrument with the DASP method characteristics. Moreover, the IRT results presented good properties about the tasks applied in our investigation (i.e., Clown task and HTKS task), reinforcing the relevance of using everyday material that is available in any common social context of learning in assessment periods. Also, meeting daily preschool practices, the interactive dynamic between the facilitator and the child stimulated with the DASP method proved to be a very common exercise (Silva Moreira and Veiga Simão, 2019), and therefore, we consider that the method interview format can provide great flexibility and authenticity to preschool assessment approaches.

As already said, the SRL framework, as a multidimensional concept, is very difficult to assess in a reliable sense. Adding to this fact, the specificities of the early childhood development represent an extra challenge to the investigation projects. Although some published instruments have been attempting to overcome obstacles such as the sparse language, and the difficulty to identify cognitive and metacognitive skills of preschool children, they keep underlying the need to design, adapt and validate instruments that both fit children characteristics and ecologic approaches (McClelland and Cameron, 2012; Perry, 2019). Previous studies used interviews to capture the on-going processes while the children were engaging in a task (Nader-Grosbois et al., 2008; Romera, 2003); others were mainly focused on one SRL phase, relying on self-report measures (Marulis et al., 2016); and others, relying on observation methods, tried to overtake self-report limitations (Dörrenbächer and Perels, 2018; Whitebread et al., 2009). However, according to our considerations, none were able to capture the SRL competencies of the preschool children solving a task in such a complete sense than the DASP method seemed to do. By validating this method, we can offer a complete measure to gather data from different sources that allow to understand, assess and intervene in the SRL process as a whole with preschoolers. Moreover, we worked with a time-friendly interview protocol and authentic tasks, underlying the ecological validity of the research, and approaching an investigation instrument to daily school practices. For those reasons, we claim that the DASP method is an innovative instrument that answers a set of needs in terms of assessing the SRL strategies of preschool children. Its potentialities analyzed through this investigation allow us to list some suggestions for practice.

### 4.3 Practical implications

In this study we have argued that the DASP method enables researchers and practitioners to capture the multidimensionality of the SRL process in an on-going process, concerning the questions posed to children. Moreover, it can be applied with different tasks and for several educational purposes (e.g., baseline, monitoring, intervention programs, etc.). The fact that the method use authentic preschool tasks makes the approach meaningful to children and provides an ecological validity to the approach (McClelland and Cameron, 2012; Perry, 2019). Therefore, its applicability is promising, both in research and educational contexts. In the educational field, the facilitator's role can be played by a preschool teacher, an educational psychologist, a special need teacher, or even a parent, as long as the SRL concept is mastered. In fact, we consider that a multidisciplinary approach is the most effective way to lead young children to get familiar with SRL strategies and practices. Furthermore, the opportunity to get reciprocal feedback in the interaction between the child and the adult, helps the first to develop SRL strategies, and the second to customize the approach to individual learning needs. In terms of research, the DASP method seems to be an appropriate instrument to collect repeated measures, allowing to design longitudinal interventions (Azevedo, 2020), and monitoring children learning progress. Also, the game-like format interview adapted to preschoolers presented indicators to be user friendly, fast and reliable to fill in, reinforcing its adequacy for research-practice in preschool context.

### 4.4 Limitations and future directions

Although some methodological limitations were overcome in our study with data triangulation, the sample was relatively small and unrepresentative. Additionally, the absence of video recordings for analysis should also be noted. So, some aspects of data collection can be improved in future studies with the audio/video recording like others did (Dörrenbächer and Perels, 2018), allowing a deeper analysis of the children's performance that may not be detected by the facilitator when conducting the interview and taking notes at the same time. Upcoming research could allow children to observe their performance, benefiting from the potentialities of a stimulated recall technique, rarely used with preschoolers (e.g., Morales and Rumenapp, 2017). We suggest including the DASP method in preschool teachers' special training and professional development, so they can practice SRL assessment and promotion in preschool daily practices (Silva Moreira and Veiga Simão, 2019). Lastly, even though this instrument may contribute to some advance in the research field, more instruments with close objectives to the DASP method should be designed to better meet the actual needs in the preschool context.

## 5 Conclusions

In recent decades, the literature has increasingly disseminated research on young children's SRL by using child-friendly instruments. However, instruments that consider the SRL concept as a whole in an on-going process and follow an ecological approach specially designed for preschool children, as adopted in this study with the DASP method, are still scarce.

The present study provides answers to its research questions, namely, RQ 1 - Will preschool children face difficulties in the different self-regulated learning phases? And, if so, what difficulties emerge in each phase? by carefully analyzing, through a cross-validation approach, how preschoolers perform, report and feel in three SRL phases, particularly, in terms of their difficulties. By applying an innovative statistical option, not often used with preschool tasks, where the instrument's properties were analyzed according to the respondents, the precision of the method in relation to the participants seems to have been reinforced. Moreover, it was possible to study the potentialities of the DASP method, as defined in RQ 2 - Can an ecological, dynamic and multidimensional approach assess overt and covert self-regulated learning processes and strategies in preschool children? showing that the multimethod approach presented herein may be an answer to the aforementioned research difficulties, and have consistent indicators on the assessment of children's emerging regulatory competence, with appropriate opportunities and authentic tasks to exercise SRL.

## Declarations

### Author contribution statement

Janete Silva Moreira: Conceived and designed the experiments; Performed the experiments; Analyzed and interpreted the data; Contributed reagents, materials, analysis tools or data; Wrote the paper.

Paula Costa Ferreira; Ana Margarida Veiga Simão: Conceived and designed the experiments; Analyzed and interpreted the data; Contributed reagents, materials, analysis tools or data; Wrote the paper.

### Funding statement

This work was supported by The Portuguese Foundation for Science and Technology of the Science and Education Ministry of Portugal [SFRH/BD/137715/2018], and by the Research Center for Psychological Science of the Faculty of Psychology, University of Lisbon (CICPSI; UIDB/04527/2020 and UIDP/04527/2020).

### Data availability statement

Data will be made available on request.

### Declaration of interest's statement

The authors declare no conflict of interest.

### Additional information

No additional information is available for this paper.

## Figures and Tables

**Table:** 

References	Advantages	Disadvantages
Al-Hroub and Whitebread (2019)	It uses a dynamic assessment to identify learners' strengths and weaknesses.	The sample includes children who are older than preschoolers.
Stevenson et al. (2013)	It clearly defines the dynamic assessment approach applied within the study. Use of an innovative approach for a demarcated data analysis (Item Response Theory).	The studies focus on the use of digital tools; low reliability as an ecologic measure to use in real preschool contexts.
De la Fuente and Lozano (2011)	Although not assuming a demarcated dynamic assessment approach, the authors applied procedures that fairly assess SRL processes (forethought, performance and self-reflection phases).	
Romera (2003)		A small sample was used.
Marulis et al. (2016)	A valuable effort to assess metacognitive awareness in preschoolers through an interview technique.	The instrument captures only a part of the SRL process.

**Table:** 

	The DASP method protocol				
	ID Child: Task: Observation beginning - Hour:				
	Show the activity to the child and explain the instruction.				
	***1. What do you think you need to do?***				

	**Category**		**Answer**		**Code**
FORETHOUGTH PHASE	Goal identification		The child identifies the goal. I have to…		3
			The child asks for help or asks to confirm the goal. Can you repeat? Should I do the same as this one?		2
			The child does not identify the goal or gives an irrelevant answer. I don't know. I forgot what I should do.		1
	***2. Now, complete the sentence: I am going to need…***				
	Organizing and transforming		The child anticipates resources and strategies. A pencil and to pay attention.		4
			The child anticipates only strategies. To be focused. To see how it should be done and then repeat. To copy the other one.		3
			The child anticipates only resources. A pencil. Some pens. My body. Your help.		2
			The child does not anticipate, or gives an irrelevant answer; I don't know.		1
	***3. How are you going to do the activity?***				
	Establishment of performance goals		The child describes the action. First…then…		3
			The child points or tells the goal in a non-verbal way. I am going to do like this one.		2
			The child does not describe or anticipate the action or gives an irrelevant answer. I don't know.		1
	***4. Do you think you can do this activity even if you are not familiar with it?****(Why?)*				
	Self-efficacy perception		☺ Yes		3
			More or less		2
			☹ No		1
	***5. Do you think it is going to be interesting, funny?****(Why?)*				
	Interest of the task perception		☺ Yes		3
			More or less		2
			☹ No		1
	***6. Do you think it is going to be easy to do this activity?****(Why?)*				
	Perception of the task's difficulty		☺ Yes		3
			More or less		2
			☹ No		1

	**Category**		**Observation**		**Code**
PERFORMANCE PHASE	Attention focus		The child maintains his/her attention on the task. Keeps the attention on and is committed to the task.		3
			The child interrupts the procedure. Directs the attention to something else. Makes comments about what is happening somewhere else.		2
			The child avoids the task. I don't know. I don't want to do it.		1
	Self-instruction		The child uses audible self-speech. The child verbalizations can be heard.		2
			The child does not use audible self-speech. The task is solved in silence.		1
	Performance strategies	Resource management and monitoring	The child manages some resources towards the goal. Is right here. Now, I need…		2
			The child does not manage resources.		1
	Performance strategies	Social assistance	The child asks for help. Can you help me? Am I doing well?		2
			The child does not ask for help.		1
	***1. Can you explain to me how you did it?***				

	**Category**			**Answer**	**Code**
SELF-REFLECTION PHASE	Self-judgement	Descriptive assessment		The child mentions specific aspects of the task performance First… then, I put… after that, I did…	3
				The child mentions general aspects of the task performance. I did like I should.	2
				The child does not describe the performance or gives an irrelevant answer. I don't know. I forgot how I did it.	1
	***2. Why did you do it this way?***				
	Self-judgement	Strategic approach assessment		The child names strategies to perform the task, achieving the initial goal. To do the same. To do like you said to me.	3
				The child names strategies to perform the task, achieving other goal. Because is easier. I've made it! I wanted to do like this.	2
				The child does not name strategies or gives an irrelevant answer. I don't know. I forgot why I did this way.	1
	***3. Did you do it well?***				
	Self-judgement	Efficacy assessment		☺ Yes	3
				More or less	2
				☹ No	1
	***4. Why?***				
	Self-judgement	Causal attribution		The child names internal causes to the result. Because that is how I know it. I was hurry.	3
				The child names external causes to the result. I hadn't everything I needed.	2
				The child does not names causes to the result. I don't know. I forgot.	1
	***5. Was it easy to do this activity?****(Why?)*				
	Self-judgement	Perception of the task's difficulty		☺ Yes	3
				More or less	2
				☹ No	1
	***6. Did you enjoy the activity?*** (*Why?*)				
	Self-reaction	Affective reaction		☺ Yes	3
				More or less	2
				☹ No	1
	***7. Are you happy with what you did?****(Why?)*				
	Self-reaction	Self-satisfaction		☺ Yes	3
				More or less	2
				☹ No	1
	**8. In the next time, how are you going to do this activity?**				
	Self-reaction	Adaptive/defense inferences		The child names cognitive or metacognitive strategies to improve the result. I am going to do better. I will pay much attention. I am going to do it well. Like you will say to me.	4
				The child identifies mistakes. This part is not like that.	3
				The child does not make changes. The same.	2
				The child does not respond or gives an irrelevant answer. I don't know.	1
	Observation end - Hour:				
